# Adoption of AI-Enabled Tools in Social Development Organizations in India: An Extension of UTAUT Model

**DOI:** 10.3389/fpsyg.2022.893691

**Published:** 2022-06-20

**Authors:** Ruchika Jain, Naval Garg, Shikha N. Khera

**Affiliations:** University School of Management and Entrepreneurship, Delhi Technological University, Rohini, India

**Keywords:** collaboration, artificial intelligence, AI aversion, UTAUT, social organizations

## Abstract

Social development organizations increasingly employ artificial intelligence (AI)-enabled tools to help team members collaborate effectively and efficiently. These tools are used in various team management tasks and activities. Based on the unified theory of acceptance and use of technology (UTAUT), this study explores various factors influencing employees’ use of AI-enabled tools. The study extends the model in two ways: a) by evaluating the impact of these tools on the employees’ collaboration and b) by exploring the moderating role of AI aversion. Data were collected through an online survey of employees working with AI-enabled tools. The analysis of the research model was conducted using partial least squares (PLS), with a two-step model – measurement and structural models of assessment. The results revealed that the antecedent variables, such as effort expectancy, performance expectancy, social influence, and facilitating conditions, are positively associated with using AI-enabled tools, which have a positive relationship with collaboration. It also concluded a significant effect of AI aversion in the relationship between performance expectancy and use of technology. These findings imply that organizations should focus on building an environment to adopt AI-enabled tools while also addressing employees’ concerns about AI.

## Introduction

The last decade has witnessed an increase in the use of artificial intelligence (AI)-based tools in organizations across all industries (Andronie et al., 2021a; Kovacova and Lăzăroiu, 2021; Nica et al., 2022). AI is used for different processes, such as forecasting future demands of products (Kawaguchi, 2021), hiring employees (van Esch et al., 2019; Li et al., 2021), formulation of marketing strategy, channel management, team management, and performance management (Davenport et al., 2020; Huang and Rust, 2020; Seeber et al., 2020a; Chatterjee et al., 2021; Nica et al., 2021; Vlačić et al., 2021). In social development organizations, employees are dispersed across locations for the intervention and execution of programs. AI-powered online tools support employees in their collaboration and task completion. Some online tools used are Slack, Microsoft Teams, Asana, Trello, and Yammer (Azarova et al., 2020; O’Connor, 2020). Utilizing its computational power, AI can collect, analyze, synthesize, predict, and identify patterns of team behavior (Valentine et al., 2017; Kellogg et al., 2020). These tools increase team efficiency using smart scheduling, auto-composing messages, voice-activated controls, virtual digital assistants, self-help desks, project management, resource allocation, and other automation that contribute to the workspace (Bousman, 2019; Dolata et al., 2019; Popescu et al., 2021). Chatbots in these AI-powered tools can monitor the chats and prompt groups to take a poll to decide on the next step (Zhou et al., 2018) or even indicate the challenges in team hierarchy and workflow (e.g., DreamTeam, Chorus.ai, etc.) (Kellogg et al., 2020). These AI-powered tools build both synchronous and asynchronous forms of communication between members for coordination, cooperation, and management, developing collaboration with greater flexibility for dispersed teams working in different regions (Maruping and Magni, 2015; Toxtli et al., 2018; Rysavy and Michalak, 2020; Lăzăroiu et al., 2021; Rogers and Zvarivoka, 2021).

In India, the use of AI-enabled tools is still in its infancy stage (Rao et al., 2021). Their use in social development organizations is also not well understood (Bajpai and Wadhwa, 2021, p. 5; Okolo et al., 2021; Albanna et al., 2022). The exposure of employees working in the social development sector to AI is also limited (Beede et al., 2020). With the increased use of technology and government initiatives, India can see a wave of transformation and the introduction of AI across the organization in social development (Bajpai and Wadhwa, 2021, p.7; Chatterjee et al., 2021). Researchers from various fields are studying the adoption and use of AI-enabled products in India (Sobti, 2019; Chatterjee et al., 2021; Jain et al., 2022). AI is expected to provide lucrative benefits, but only if contributions are translated into actions (Brown and Sandholm, 2018; Nica and Stehel, 2021). Thus, organizations are exploring how to best increase employee acceptance of AI-enabled tools across domains and industries (Sharma, 2020; Chatterjee et al., 2021; Rana et al., 2021; Al-Nuaimi et al., 2022). Based on the identified future challenges with AI, this study aims to understand the factors that influence the adoption of AI-enabled tools by employees in social development organizations. It also evaluates the impact of these tools on employees’ perceived experiences.

It is important to note that the potential benefits of AI can only be realized if employees accept its use (Logg et al., 2019; Andronie et al., 2021b). The use of AI has always been an antagonist (Duan et al., 2019) and controversial phenomenon (Hou and Jung, 2021). Despite the algorithm’s accuracy, researchers and practitioners have witnessed people’s reluctance to use algorithms (Burton et al., 2019; Mahmud et al., 2022). This rejection is referred to as algorithm aversion (Dietvorst et al., 2015). It is defined as “a behavior of neglecting algorithmic decisions in favor of one’s own decisions or other’s decisions, either consciously or unconsciously” (Mahmud et al., 2022, pp. 1). The employees’ negative perception of AI can be attributed to the fear of job substitution, a lack of training, uncertainty (Frey and Osborne, 2017), a poor understanding of how to use AI (Raisch and Krakowski, 2020), and a lack of trust in AI systems (Glikson and Woolley, 2020). These factors inhibit the integration of employees and AI. Employees in the social development sector may have limited or no understanding of technology, making it difficult for organizations to adopt AI-based tools.

The aversion toward AI can be due to its distinction from other forms of technology (Puranam, 2021). Venkatesh (2021) urged to identify unique antecedents in adopting AI-based technology with its emerging form. Researchers have been encouraged to look beyond traditional technology adoption models to understand the contextual condition and the attributes unique to these emerging technologies (Brown et al., 2010; Hong et al., 2014; Dwivedi et al., 2019, Dwivedi et al., 2020). Social development organizations need to identify employee-specific antecedents that drive the adoption of AI-powered tools (Kaur and Arora, 2020; Dabbous et al., 2021). Many studies evaluating technology acceptance models have been criticized for their approach, for limiting their measures to use or intention of use (Venkatesh, 2021). There is a need to expand the model to see the impact of introducing this new technology to end-users. Extending the model will aid in understanding this social and technological convergence (Bednar and Welch, 2020; Makarius et al., 2020).

Adoption and use of AI-enabled features are entirely voluntary, and employees are free to use them to assist the team in task completion and improve team decisions. However, even with AI’s potential to enhance employees’ ability to effectively develop and enhance team collaboration (Haas and Mortensen, 2016; Daugherty et al., 2019; Webber et al., 2019), there is still limited applicability of it in understanding team experiences (Larson and DeChurch, 2020; Zhang et al., 2021). AI provides support to team functions with information that can help teams collaborate better (such as conducting team polls, analyzing the chats, team participation, and so on.); however, their input and functions are not readily adopted by employees working in a team (Chatterjee et al., 2020; Sharma, 2020; Murray et al., 2021). Past literature suggests that the nature of collaboration and how members interact with a team changes with the introduction of AI, as that affects team experiences (Shamekhi et al., 2018; Webber et al., 2019; Larson and DeChurch, 2020; Seeber et al., 2020b; Hopkins, 2022). Contemporary researchers are exploring factors that influence the adoption of AI by employees in organizations (Pumplun et al., 2019; Alsheibani et al., 2020, Alsheibani et al., 2020; Pelau et al., 2021), but there is less evidence of how these AI-powered interactive tools affect the workforce and improve collaboration (Frommert et al., 2018; Waizenegger et al., 2020). This study makes three major contributions. First, it identifies the role of social influence, performance expectancy, facilitating condition, effort expectancy, and AI aversion in adopting and using AI-based tools in social development organizations. Second, it highlights the role of AI aversion in adopting and using these tools. It also highlights the role of social influence and facilitating conditions in addressing AI aversion and how its presence influences the relation between performance expectancy and use. Finally, the study demonstrates the role of AI-enabled tools in building collaborative experiences for employees.

Extending on the UTAUT model, this study used the research model to analyze 415 responses from employees working in a social development organization in India that use AI-enabled tools for team interaction and task management. The empirical evidence supports the model, and its association provides practical implications. The findings propose developing a favorable social influence for the employees to adopt AI-based collaboration tools. Organizations should make a conscious effort to address employees’ concerns about AI-centered technology and help to address their concerns. The rest of the paper is structured as follows: Section 2 presents a brief overview of human-AI collaboration and UTAUT models for understanding AI adoption. The research hypothesis is developed in Section 3. Section 4 includes the research methodology. Following this, Section 5 elaborates on the analysis and results. Sections 6, 7, and 8 discuss findings, implications, and research limitations. Finally, Section 9 has the conclusion.

## Review of Literature

### Human Artificial Intelligence Collaboration

Artificial intelligence-based tools are used for efficient team collaboration and task completion (Kellogg et al., 2020). The technological advancement in AI allows humans to use it as a collaborator for diverse knowledge-intensive tasks (Seeber et al., 2020b). AI facilitates human thinking and problem-solving with increased efficiency in organizations (Malone, 2018; Wilson and Daugherty, 2018). This augmentation can help to balance humans in making unbiased judgments, better decisions, greater creativity, and bounded rationality in finding solutions (Kanhemann et al., 2016; Burton et al., 2019). Machine and human inputs are combined to make decisions greater than their individual decision-making abilities (Kamar, 2016; Wang et al., 2016). However, according to a Deloitte report (2017), a survey of senior managers working on more than 150 AI projects found it challenging to integrate AI with existing people, processes, and systems. Studies have highlighted the gap in the application of AI in manager roles (Henke and Kaka, 2018) due to the limited understanding of the interplay between humans and AI (Traumer et al., 2017; Kellogg et al., 2020). The potential benefits of human-AI collaboration can only be realized when employees in the organization trust, accept, and use technology for work processes (Caputo et al., 2019; Wamba-Taguimdje et al., 2020; Chowdhury et al., 2022). The literature points to two areas of concern with AI (1) the negative impact of AI such as biases, discrimination, and bad decisions (Davenport et al., 2020) and (2) the possibility of losing their jobs (Rampersad, 2020). The apprehension toward AI can be non-factual, with the perceived threat like when the AI contradicts people’s judgment (Elkins et al., 2013) or a lack of knowledge by humans of AI (Mahmud et al., 2022). The recent work of Makarius et al. (2020), using the tenants of socio-technical system theory and organizations socialization theory, proposes integrating AI as another employee in an organization. The work of Chowdhury et al. (2022) on employee-AI collaboration has urged the need to explore context-specific factors and bring emerging constructs and proxies to measure human-AI collaboration for business performance. It is essential to understand that the problem has shifted from the AI application to understanding factors driving and inhibiting AI-employee integration (Fleming, 2019; Haenlein and Kaplan, 2019; Makarius et al., 2020, Murray et al., 2021). Some of the recent works are exploring factors that influence the use of AI-enabled tools in organizations (Al Shamsi et al., 2022; Al-Sharafi et al., 2022). It is acknowledged that AI research is proliferating in its business value. However, there is a gap in empirical research on achieving human-AI integration in the organization. There is a lack of a theoretical framework to understand employee and AI collaboration (Chowdhury et al., 2022).

### Unified Theory of Acceptance and Use of Technology

Unified theory of acceptance and use of technology model is a widely used theory to study the adoption of technology (Al-Qaysi et al., 2021). This theory extends Brown et al.’s (2010) collaboration theory, evaluating the role of technology in both initial and post-adoptive stages (Venkatesh et al., 2011). The model aims to explain the use of technology and user behavior under voluntary conditions (Venkatesh et al., 2003). There are four predictors of the use of technology: performance expectancy, effort expectancy, social influence, and facilitating condition (Venkatesh et al., 2003). Dwivedi et al. (2019) have studied the revised UTAUT model where the four exogenous constructs are viewed as representing technology attributes (performance expectancy and effort expectancy) and contextual attributes (social influence and facilitating conditions). Previous research has shown that the UTAUT model is robust in explaining a high degree of variance (70%) in users’ intention to use technology. Still, the traditional model of UTAUT has been found insufficient to study AI, as it only focuses on the use of functional techniques and cannot explain complex processes involved in AI adoption (Gursoy et al., 2019). While using the UTAUT model, many studies have limited their focus by evaluating technology’s success in terms of performance and user satisfaction (Montesdioca and Maçada, 2015). There is a need to assess the outcome of these technologies on individuals.

The model has been used with some emerging technologies with AI. Gursoy et al. (2019) evaluated the UTAUT model to assess the willingness of the consumer to use AI devices. They found that social influence, hedonic motivation, and anthropomorphism influenced performance expectancy and effort expectancy, which further influenced emotions and impacted the willingness to use devices. In operation management, the adoption of AI use was influenced by six factors, including social factors and facilitating conditions (Grover et al., 2020). Chatterjee et al. (2021) evaluated the use of AI-integrated customer relation systems in Indian organizations, and their findings validated the models underpinning and other exogenous customer relationship management variables. Chatterjee and Bhattacharjee (2020) assessed the adoption of AI in higher education, where perceived risk, facilitating condition, and effort expectancy impact attitude, influencing the intention to use AI in higher education. Cao et al. (2021) extended the model with the development of an integrated AI acceptance–avoidance model, which considers positive and negative factors that influence managers’ attitudes and behavior toward using AI. Studies have acknowledged the negative influence of technology (Agogo and Hess, 2018; Vimalkumar et al., 2021). Thus, the negative perception of AI must be considered in developing a model (Davenport et al., 2020).

## Proposed Conceptual Model and Hypothesis Development

### Performance Expectancy

Performance expectancy is the extent to which users believe that technology use will help them satisfy their job-related needs (Venkatesh et al., 2003). This study identifies it as the employees’ belief that AI-enabled tools will support their job performance and teamwork. The past literature has shown a strong association between performance expectancy and the use of technology (Brown et al., 2010; Hong et al., 2011; Wang et al., 2014), and extending it to AI-enabled tools would only be logical. Studies have shared the association of AI with increased performance (Ransbotham et al., 2018; Duan et al., 2021), which can be perceived as an association with performance expectancy (Cao et al., 2021). Contemporary researchers have shown a clear association between performance expectancy and effort expectancy with AI use (Gursoy et al., 2019; Lin et al., 2020). The extant literature shares a strong proposition of AI aversion that influences the adoption of AI (Dietvorst et al., 2015). However, there is a limited empirical measure of this association in information technology research models (Cao et al., 2021). Cao et al. (2021) found no significant association between performance expectancy and intention to use technology, but a positive influence was found on attitude toward AI in decision-making. Chatterjee et al. (2021) found that performance expectancy was associated with using AI-enabled systems to enhance performance. Based on the above review, the following hypotheses were developed:

H1: Performance expectancy will positively affect the use of AI-enabled tools.

H9: AI aversion will mediate the influence of performance expectancy and the use of AI-enabled tools.

### Effort Expectancy

Effort expectancy is defined as the degree of ease with which one can operate a system (Venkatesh et al., 2003). Several studies have proposed the antecedent role of effort expectancy in technology adoption (Martins et al., 2014; Mutahar et al., 2016). Davis (1989) support this, as users at a cognitive level would understand this as a trade-off between the effort required to apply the technology and the benefits achieved by using it. Cimperman et al. (2016) found that effort expectancy is an antecedent, including complexity and ease of use in technology adoption. This research identified it as the ease or difficulty of using AI-enabled tools for team activities. In their review, Dwivedi et al. (2019) explained effort expectancy as a technological attribute in the use of AI. They found that effort expectancy strongly influences attitude, which mediates the influence of using technology (Chatterjee et al., 2021). The complex design and low explainability of outcomes in using AI-based tools can influence the adoption of AI in India (Cheatham et al., 2019; Dwivedi et al., 2021). Technology that can ease the effort required to accomplish a task is more likely to be used. Based on this review, the following hypotheses were developed:

H2: Effort expectancy positively influences the use of AI-enabled tools in the organization.

H10: AI aversion will mediate the influence of effort expectancy and the use of AI-enabled tools.

### Facilitating Conditions

Facilitating conditions are the extent to which the user believes that adequate support and resources are available for using technology in organizations (Venkatesh et al., 2003). In the present research, technical and organizational support provided to employees to adopt and use online AI-enabled tools in their tasks is considered facilitating conditions. Lee et al. (2013) found that facilitating conditions determine the acceptance and use of innovative technology. To ensure technology is utilized, these conditions must be introduced well to users (Guo, 2015) as they play a critical role in influencing user behavior toward technology (Guo, 2015; Chatterjee et al., 2021). A recent meta-analysis of the UTAUT model found that facilitating conditions also play a role in developing a positive attitude toward using technology (Dwivedi et al., 2019). Studies regarding AI use have found a positive relationship between facilitating conditions and behavioral intention to use AI (Chatterjee and Bhattacharjee, 2020). Past literature supports facilitating conditions over performance expectancy (Rana et al., 2016) and effort expectancy (Dwivedi et al., 2017, Dwivedi et al., 2019). In India, it was found that government support in the form of training, a facilitating condition, had a positive impact on performance expectancy, increasing the adoption of electronic government systems in selected Indian cities (Rana et al., 2016). Recent works have emphasized the impact of facilitating conditions on AI-specific attitudes in specific technological structures (Dwivedi et al., 2021), organizing designing policies, procedures, and training (Cheatham et al., 2019), as they improve perception toward AI leading to its adoption. The literature helps us to develop the following hypotheses:

H3: Facilitating conditions positively influence the usage behavior of AI-enabled tools in organizations.

H6: Facilitating conditions influence algorithmic aversion.

### Social Influence

Social influence is defined as how users’ perception of technology is influenced by their social environment (Venkatesh et al., 2003). In this research, the environment for social influence comprised of peers, seniors, and management, and how they influence the use of AI-enabled tools in organizations. In the model, it has been proposed that social influence on user behavior could result from compliance, especially during the initial use of technology (Venkatesh et al., 2003). Past studies have found that social influence impacts the use of technology in different contexts. It plays a prominent role in using technology in mandatory and voluntary settings (Gupta et al., 2008; Gruzd et al., 2012). Studies have shared the roles of co-workers and supervisors in using technology (Cheng, 2011; Brown et al., 2014). Dwivedi et al. (2019) emphasized social influence as a contextual factor in using technology. They proposed using social influence to develop a positive attitude toward technology. Considering the case of AI-enabled tools used by teams to manage work, social influence can play a critical role in using technology, as employees might fear missing out (van Esch et al., 2019). In the context of AI services, Gursoy et al. (2019) shared how social influence is a crucial antecedent to predicting the use of technology. Based on the above review, we would like to test the following hypotheses:

H4: Social influence positively influences the use of AI-enabled tools in organizations.

H7: Social influence impacts algorithmic aversion.

### Algorithmic Aversion

While there is great appreciation for the potential of AI to revolutionize organizations, there is still some uncertainty about its consequences on people and organizations (Bedué and Fritzsche, 2021). Frick (2015) said, “as these machines evolve from tools to teammates, one thing is clear: Accepting them will be more than simply adopting new technology.” In collaborative initiatives, the acceptance or avoidance of AI is dependent on the perception of AI. Early literature on algorithmic support suggests that people avoided inputs from algorithms even when they had information about superior performance (Dietvorst et al., 2015; Prahl and Van Swol, 2017). There are different situations and domains where this phenomenon has been found (Bigman and Gray, 2018; Castelo et al., 2019). Researchers have also shown that algorithmic aversion is exhibited even when the accuracy of AI is identical to that of humans (Berger et al., 2020; Bogert et al., 2021). The level of awareness about the expertise and efficiency of algorithms also influences algorithmic aversion (Bigman and Gray, 2018; Zhang et al., 2021). The lack of knowledge about algorithm use hinders its usage (Mahmud et al., 2022). There is an aversion toward AI compared to human experts’ decisions and one’s own decisions (Litterscheidt and Streich, 2020; Stein et al., 2020; Kawaguchi, 2021). Studies have also discovered that some people are intrinsically averse to AI, irrespective of their performance, due to fundamental distrust of algorithms (Prahl and Van Swol, 2017; Kawaguchi, 2021). Avoiding AI tends to undermine usage, thus inhibiting human-AI collaboration. Recent systemic reviews have revealed a gap in the firm-level analysis of the adoption of AI (Mahmud et al., 2022). They emphasized that adoption is a macro-level activity since using the algorithm is a firm activity. Only recently, there has been a study on understanding firm-level analysis of factors influencing managers’ attitudes on using AI for decision-making (Cao et al., 2021). Adopting AI-based inputs for team activities can be the employee’s choice. Their aversion toward technology can influence its adoption in social development organizations in India. Based on the above review, the following hypothesis is developed:

H5: Algorithmic aversion influences the use of AI-enabled tools.

### Outcome Variable

The present research identifies the outcome variable as the collaboration climate experienced by employees using AI-enabled tools to manage tasks and members. Many studies have investigated the use of technology as an outcome (Raza et al., 2020; Chatterjee et al., 2021). There is a prominent gap in the literature regarding the impact of technology on different outcome variables (Venkatesh, 2021). The use of technology can impact performance or overall benefits in the organization, and it must be measured to assess the success of technology (Montesdioca and Maçada, 2015). Earlier studies have only focused on using technology-specific outcomes of satisfaction with technology or its effect on performance (Hou, 2012). Only selected studies have focused on understanding the specific impact of technology on the quality of decision-making, job efficiency, job performance, communication quality, innovation ideas, job effectiveness, and work-life quality (Isaac et al., 2019). Many tools claim their influence on effective collaboration in teams and management of tasks. However, there is minimal empirical literature that assesses the collaborative experience of employees with the use of AI-enabled tools in groups (Larson and DeChurch, 2020). Researchers have identified human-AI collaboration as teammates as a challenge (Seeber et al., 2020a). Some recent studies explore effective ways for human-AI collaboration in organizations by developing frameworks (Makarius et al., 2020) and identifying antecedents essential for effective integration (Chowdhury et al., 2022). This study investigates the effects of AI usage on employees’ and organizations’ benefits, contributing to the research gap in understanding the impact of AI-enabled tools on the collaborative experiences of employees.

H8: The use of AI-enabled tools positively influences the collaborative climate in organizations.

### Research Model

**FIGURE 1:**
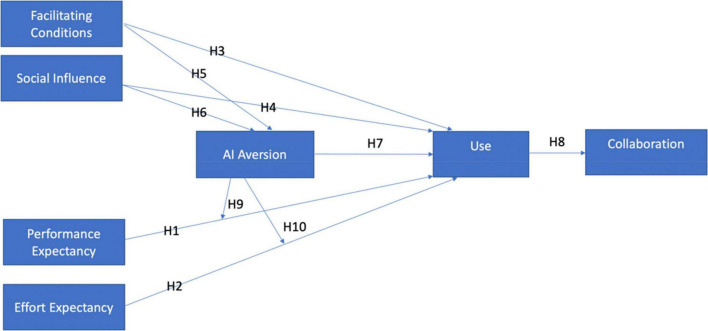
Proposed research model. Source: primary data.

The UTAUT model of Venkatesh et al. (2003) provides all relevant factors that help to determine employees’ use of AI and its impact on organizations. Recent work with the model has suggested extending its evaluation beyond behavioral intention and the use of technology (Venkatesh, 2021). The model extends with the inclusion of the AI aversion model to assess its influence on adoption. Based on the identified factors, the proposed model encourages AI-enabled tools in organizations. Further, it presents an understanding of their impact on the collaboration experiences of employees in organizations (Figure 1).

## Research Methodology

### Sample and Data Collection

To test the hypotheses, we collected data using an online survey from social development organization that actively used AI-enabled tools. A purposive sampling technique was used to target middle and senior managers in social development organizations who actively used these tools to collaborate with their distributed teams. The employees below the managerial level were excluded as they did not use the tools. The sample size was determined based on the expected R2 value of the constructs (Hair et al., 2014). Because the model allows for a maximum of five arrows pointing toward the construct, 205 responses were required to detect a minimum value of 0.10 at a significance level of 1% (Hair et al., 2014). To ensure potential non-response bias, Armstrong and Overton’s (1977) procedure was undertaken. Independent sample *t*-test and chi-square test were performed on the first 150 and last 100 respondents. There was no deviation in results for the two groups, thus confirming that the responses are free from non-response bias. A total of 412 responses were collected. On analysis of the result, twenty-four responses were discarded (14 due to missing data, 5 as outliers, and 5 in the straight-lining). The sample comprised of 60.5 men and 39.4% women. Also, employees in 18– 24-, 25– 34-, 35– 44-, 45– 54-, and above 55-year age categories were 111.8, 36.5, 36.3, 13.6, and 1.5%, respectively. The detailed demographic description of the sample is reported in Table 1.

**TABLE 1:** Demographic description of sample.

Variable	Category	Frequency	Percentage
Gender	Male	235	60.5%
	Female	153	39.5%
Age	18–24 Yrs	46	11.8%
	25–34 Yrs	142	36.5%
	35–44 Yrs	141	36.3%
	45–54 Yrs	53	13.6%
	55 and above Yrs	6	1.5%
Experience	0–5 Yrs	153	39.4%
	6–10 Yrs	91	23.4%
	11–15 Yrs	76	19.5%
	16–20 yrs‘	41	10.5%
	Above 20 Yrs	27	6.9%

*Source: primary data.*

## Measures

### Performance Expectancy, Effort Expectancy, Social Influence, and Facilitating Conditions

These variables were measured using an adapted version scale developed by Venkatesh et al. (2012). This adapted version was validated by Martins et al. (2014). It is a five-point rating scale ranging from 1 (strongly agree) to 5 (strongly disagree). A few items on the scale are “using AI-enabled tools to have faster communication with my team” (performance expectancy, 4 statements), “my interaction with AI-enabled tools is clear and understandable” (effort expectancy, 4 statements), “people who influence my behavior think that I should use AI-enabled tools” (social influence, 3 statements), and “AI-enabled tools are compatible with other technologies I use” (facilitating conditions, 4 statements).

### User Behavior

User behavior was assessed with the help of an adapted version of the three-item scale by Ain et al. (2016). Considering the use of AI as an anxiety-producing phenomenon, the scale was adapted to measure aversion toward AI (It was also a five-point rating scale ranging from 1 (strongly agree) to 5 (strongly disagree). A few scale statements are “I depend on AI-enabled collaborative tools” and “I use many functions of AI-enabled tools.”

### Artificial Intelligence Aversion

The aversion toward AI was measured using an adapted version of the fear-based xenophobia scale (van der Veer et al., 2013). The reliability coefficient for the scale was found to be 0.87. One of the items included was “Interacting with and using AI makes me uneasy.” The scale was measured using a 1–5 Likert scale.

### Collaboration Climate

The scale was assessed with the help of the adapted version of the 14-items collaboration climate scale developed by Chiocchio et al. (2012). It had a five-point rating scale ranging from 1 (strongly agree) to 5 (strongly disagree). A few scale statements are “While using online tools, my teammates and I provide each other with useful information that makes work progress” and “While using online tools, my teammates and I understand each other when we talk about the work to be done.” All measures are presented in Supplementary Table 1.

## Results and Data Analysis

The research model was analyzed with the help of the partial least squares (PLS) method using SmartPLS software (version 3.0) (Ringle et al., 2015). In recent work, PLS-SEM is an emerging approach in social and behavioral discipline (Hair et al., 2021). The rationale for using PLS-SEM (variance-based SEM) than covariance-based SEM (CB-SEM) is based on the following points. First, PLS-SEM is applied when the objective is to develop and predict the construct of a theory (Hair et al., 2014, 2016), as in the case of adoption of AI-enabled tools. They are found to work when the models are complex and they make no assumption with non-normally distributed data (Hair et al., 2019). Also, they are found to be a promising method to extend an existing structural theory (Hair et al., 2011; Ringle et al., 2012; Willaby et al., 2015). Second, when there is slight prior knowledge on the structural model relationship, the measurement of constructs, or when the emphasis is on exploration than confirmation, as in this study, PLS-SEM is found to be a more powerful alternative to CB-SEM. A two-stage analytical process measurement model was assessed, followed by the structural model (Anderson and Gerbing, 1988; Hair et al., 2017). PLS-SEM allows the analysis of data that is not normally distributed as in this study (Akter et al., 2017).

### Measurement Model

To avoid any misspecification, a confirmatory tetrad analysis (CTA-PLS) was conducted (Gudergan et al., 2008; Hair et al., 2013). The result found that the measurement model was reflective. The reflective measurement model was further assessed for internal reliability using Cronbach’s alpha (CA) and composite reliability (CR) values. The convergent validity was measured using average variance extracted (AVE) estimates. Table 2 presents the item loading, CA, CR, and AVE values of all study variables. The results of the measurement model indicate that loadings of items range from 0.60 to 0.94, which are greater than the recommended levels of 0.50 (Hair et al., 2010). CA and CR measures for all constructs range from 0.70 to 0.95, and these values are higher than the recommended cutoff of 0.70 (Fornell and Larcker, 1981; Hair et al., 2010). The AVE for variables was higher than 0.50, which established the convergent validity of the scale. Table 2 also revealed that the factor loading of items was greater than 0.70, concluding indicator reliability of the variables (Hair et al., 2017).

**TABLE 2:** Mean, standard deviation, loading, Cronbach’s alpha, CR, and AVE.

Construct	Item	Loading	M	SD	CA (> 0.7)	CR (> 0.7)	AVE (> 0.5)
Performance expectancy (PE)	PE1 PE2 PE3 PE4	0.755 0.727 0.742 0.756	4.274	0.62	0.81	0.83	0.55
Effort expectancy (EE)	EE1 EE2 EE3 EE4	0.754 0.731 0.818 0.744	4.880	0.52	0.80	0.84	0.58
Social influence (SI)	SI1 SI2 SI3	0.815 0.781 0.795	5.793	0.74	0.71	0.73	0.63
Facilitating conditions (FC)	FC1 FC2 FC3 FC4	0.788 0.758 0.851 0.799	5.901	0.68	0.82	0.87	0.63
Use	U1 U2 U3	0.881 0.785 0.727	4.327	0.61	0.70	0.73	0.64
Collaboration	CO1 CO2 CO3 CO4 CO5 CO6 CO7 CO8 CO9 CO10 CO11 CO12 CO13 CO14	0.817 0.716 0.789 0.860 0.748 0.887 0.751 0.772 0.718 0.728 0.872 0.831 0.741 0.794	5.14	0.38	0.91	0.95	0.62
AI aversion	AIav1 AIav2 AIav3 AIav4 AIav5	0.756 0.825 0.802 0.741 0.764	4.10	0.67	0.87	0.89	0.60

*Source: primary data, M – mean, SD – standard deviation, α – Cronbach’s alpha, CR – composite reliability, AVE – average variance extracted.*

To establish the discriminant validity of the measurement model, three different criteria were used: cross-loading, Fornell–Larcker, and hetrotrait-monotrait (HTMT) ratio. The scientific literature has established cross-loading as the primary technique to establish discriminant validity (presented in Supplementary Table 2). The results indicate that the factors fulfill the model requirement, as the outer loading among the indicator’s constructs is higher than the cross-loading value of other constructs. The second criterion to establish discriminant validity was the Fornell–Larcker test, represented by the square root value of all AVEs (Table 3). These values are higher than the correlations measured among the other constructs. These results recommend good discriminant validity of the model (Hair et al., 2017). Also, Kline (2010) suggested that the HTMT ratio of correlation values should be less than 0.85 to conclude discriminant validity. Table 4 illustrates that HTMT ratios are less than 0.85, establishing the discriminant validity of the model.

**TABLE 3:** Fornell–Larcker criterion test of discriminant validity.

Variables	PE	EE	SI	FC	Use	CO	AIav
**PE**	**0.745**						
**EE**	0.697	**0.762**					
**SI**	0.551	0.515	**0.797**				
**FC**	0.534	0.624	0.501	**0.799**			
**Use**	0.609	0.593	0.502	0.641	**0.800**		
**CO**	0.602	0.584	0.575	0.584	0.573	**0.789**	
**AIav**	0.229	0.212	0.350	0.304	0.253	0.293	**0.778**

*Source: primary data, PE – performance expectancy, EE – effort expectancy, SI – social influence, FC –facilitating conditions, CO – collaboration, AIav – AI aversion.*

**TABLE 4:** Heterotrait–monotrait ratio (HTMT).

Variables	PE	EE	SI	FC	Use	CO	AIav
PE							
EE	0.815						
SI	0.732	0.641					
FC	0.682	0.804	0.683				
Use	0.716	0.768	0.642	0.843			
CO	0.673	0.662	0.689	0.734	0.668		
AIav	0.269	0.260	0.576	0.393	0.309	0.348	

*Source: primary data, PE – performance expectancy, EE – effort expectancy, SI – social influence, FC – facilitating conditions, CO – collaboration, AIav – AI aversion.*

### Common Method Bias

The data in our study were self-reported and can have common method biases due to consistency motive and social desirability (Podsakoff et al., 2003). To address this, the study used both procedural and statistical methods. To reduce bias in the responses, anonymity and confidentiality of the respondents were maintained, as no personally identifiable information was collected, reducing the probability of providing a socially desirable response (Chang et al., 2010). The items were kept simple, easy to understand, and specific to reduce ambiguity. The statements for each construct were not grouped, and the constructs being measured were not labeled to reduce the possibility of guessing and finding links between constructs (Parkhe, 1993). The statistical method used was Harman’s single factor test (SFT). The first factor resulted in 38.5% variance, as the value was less than the highest recommended value of 50% (Podsakoff et al., 2003), concluding the presence of CMV.

### Structural Model Assessment

**FIGURE 2:**
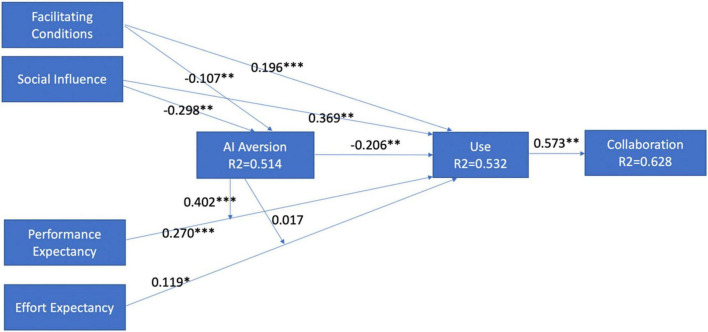
Final model. Source: primary data, ***p < 0.001, **p < 0.01, *p < 0.05.

The structural model and its relationship were evaluated in terms of collinearity, significance, and relevance (Hair et al., 2014). The model was blindfolded to ensure predictive relevance using a bias-correlated and accelerated bootstrapping procedure with 5,000 resamples. To obtain cross-validity redundancy, the omission separation was set to 5 (Henseler et al., 2014). No collinearity issue was present. The Stone–Geisser Q2 (Stone, 1974; Geisser, 1975) model value was 0.65, confirming the predictive relevance. Henseler et al.’s (2014) method was used to assess the model fit, and standard root mean square residual (SRMR) was considered. The value of SRMR was 0.059 with PLS and 0.032 with PLSc. The values were well within the highest permissible value of SRMR, i.e., 0.08 (Hu and Bentler, 1998). R2 was used to assess the model’s predictive power, i.e., the amount of variance attributed to the latent variables. The R2 values indicate that the full model showed 53% of the variance in user behavior, facilitating conditions and social influence showed 51.4% of the variance in AI aversion, and user behavior showed 63% of the variance in collaborative behavior (Table 5). Wetzels et al. (2009) shared that the effect size larger than 0.36 is considered accepted in information technology when PLS is used. The path coefficient, coefficient of determinants (R2), and VIF are shown in Table 5. The model after validation is shown in Figure 2 with all the results.

**TABLE 5:** Result of path analysis first order.

Hypothesis	Relationship	Std. Beta	Std. Error	t value	Decision	R2	VIF
H1	PE-Use	0.270	0.045	5.198***	Supported	0.532	2.179
H2	EE-Use	0.119	0.055	2.199*	Supported		2.382
H3	FC-Use	0.196	0.042	2.911**	Supported		1.592
H4	SI-Use	0.369	0.054	7.840***	Supported		1.784
H5	AIav-Use	−0.206	0.052	4.745***	Supported		1.842
H6	FC-AIav	−0.107	0.044	2.115*	Supported	0.514	1.367
H7	SI-AIav	−0.298	0.052	5.275***	Supported		2.145
H8	Use-Co	0.573	0.037	13.737***	Supported	0.628	1.000

Source: primary data, **p < 0.001, **p < 0.01, *p < 0.05, PE – performance expectancy, EE – effort expectancy, SI – social influence, FC – facilitating conditions, CO – collaboration, AIav – AI aversion.

### Hypothesis Testing

Table 5 suggests that all the hypotheses, i.e., H1 (β = 0.270, t = 5.198, *p* < 0.001), H2 (β = 0.119, t = 2.199, *p* < 0.01), H3 (β = 0.196, t = 2.411, *p* < 0.01), H4 (β = 0.369, t = 7.840, *p* < 0.001), and H5 (β = −0.206, t = 4.475, *p* < 0.001) were accepted. The results imply that effort expectancy, performance expectancy, facilitating conditions, social influence, and algorithmic aversion significantly affect the use of AI-enabled tools. Social influence has the strongest influence on using AI-enabled tools in the organization. The exogenous factors showed 53.2% of the variance in using AI-enabled tools.

The sixth and seventh hypotheses tested facilitating conditions and social influence on algorithmic aversion. Table 5 suggests that hypotheses H6 (β = −0.107, *t* = 2.115, *p* < 0.01) and H7 (β = −0.298, *t* = 5.275, *p* < 0.001) are accepted. The result shows that facilitating conditions and social influence impact algorithmic aversion. These factors showed 51.4% of the variance in algorithmic aversion. The eighth hypothesis tested how the use of AI-enabled tools influences the collaborative climate in the organization. This hypothesis is supported (β = 0.573, *t* = 13.737, *p* < 0.001). It recommended that AI-enabled tools have a strong positive influence on employees in experiencing a collaborative climate.

This study also explored the moderating role of algorithmic aversion on performance expectancy and effort expectancy. Table 6 shares the results of hypotheses H9 and H10. The results revealed that algorithm aversion significantly moderates the relationship between performance expectancy and use.

**TABLE 6:** Moderating effect.

Hypothesis	Relationship	Std. Beta	Std. Error	t value	Decision

Moderating the role of AI aversion					
H9	PE-Use	0.402	0.032	2.66	Supported
H10	EE-Use	0.119	0.043	0.59	Not Supported

*Source: primary data, PE – performance expectancy, EE – effort expectancy, SI – social influence, FC – facilitating conditions, CO – collaboration, AIav – AI aversion.*

## Discussion

This study uses the UTAUT model to identify antecedents that influence the adoption and use of AI-enabled tools in social development organizations. The model assesses the role of AI aversion in adopting these tools and the influence of the contextual attributes. Further, the model helps in studying the impact of AI-enabled tools on the collaborative experiences of employees. The results revealed that performance expectancy, effort expectancy, social influence, facilitating conditions, and algorithmic aversion influence the use of Al-enabled tools. These factors account for 53% of the variance in the usage of tools. According to UTAUT, performance expectancy positively influences the use of AI-enabled tools. This indicates that if the employee believes that the devices would support their performance, there is a greater probability that the tools would be used. Employees are more likely to adopt an integrated tool that unifies and automates various required processes to complete their work in virtual work design. These findings support the findings of previous researchers such as Andrew et al. (2021), Chatterjee et al. (2021), and Handoko and Lantu (2021), where performance expectancy influences the adoption of AI-enabled tools.

Similarly, the results suggest that effort expectancy is positively associated with the usage behavior of AI-enabled tools. They indicate that if employees perceive AI-enabled tools to be easy to use, they can adopt new technology without much effort. This result supports the findings of previous scholars such as Guo (2015), Chatterjee and Bhattacharjee (2020), and Chatterjee et al. (2021). Lin et al. (2020) studied the performance expectancy and effort expectancy in the second stage of three-step phenomena for the use of AI in the customer service industry. They assessed the influence of performance expectancy and effort expectancy on emotions while this study assessed their impact on usage. Also, this study focused on the use of AI tools by employees working in social development organizations.

The results also found a positive association between social influence and AI-enabled tools. This suggests a positive effect of peers, co-workers, and superiors on the usage behavior of employees. These results support the findings of the previous studies with other forms of technologies (Sumak et al., 2010; Martins et al., 2014). In many of the recent works with AI adoption, social influence was not considered during the evaluation of the adoption of technology for individual use such as AI for decision-making (Cao et al., 2021), adoption of CRM systems (Chatterjee et al., 2021), and educational use (Chatterjee and Bhattacharjee, 2020). Though Gursoy et al. (2019) highlighted the role of social influence in adopting AI tools, they assessed its influence on performance expectancy and effort expectancy, which further influence the emotion and use of AI-based services. According to this study, social influence has the most significant influence on the use of technology. The online tools used in an organization are for teams to work together. Their use by the employees working in teams is voluntary, and if one member adopts the use of technology, it will impact others. The social identity theory (Tajfel et al., 1979) emphasizes how adopting group behavior can strengthen an individual’s attachment level to the group. This implies that as the technology is used in a team, employees are forced to use the technology to gain greater acceptance by other group members.

Facilitating conditions also positively influence user behavior. The support provided by the organization in terms of training and infrastructure can influence employees’ usage behavior. The results of this study are aligned with those of previous studies (Chang, 2013; Chatterjee and Bhattacharjee, 2020; Chatterjee et al., 2021). Chatterjee and Bhattacharjee (2020) found facilitating conditions to positively influence behavior intention to adopt artificial intelligence in higher education. In India, considering the novelty of AI-enabled tools for employees, support from the organization is conducive to the usage of technology. Employees in social development organizations have limited exposure to technology-based resources, and building knowledge about technology and providing training can help to adopt the new technology.

The research model found a negative influence of aversion on user behavior. As the employee’s aversion toward AI increases, there is a decrease in the use of these tools. The findings align with the past literature, as the role of aversion is well-founded to influence the adoption of AI (Burton et al., 2019; Mahmud et al., 2022). In the past literature, only limited studies have included emotions (Lin et al., 2020) and perception of threats toward technology (Cao et al., 2021). Cao et al. (2021) found that personal concerns and perceived threats negatively influence attitude and intention to use technology. These findings critically impact organizations as employees’ negative perceptions of technology can be detrimental to technology adoption. AI aversion was further evaluated for its role in influencing performance expectancy and effort expectancy on their impact on use. The result found that AI aversion moderates the relation between performance expectancy and use. The aversion toward AI influences employees’ perception of increased performance using AI tools. This aversion could result from a lack of knowledge, belief in the capability of AI, or fear of job loss, which can result in avoidance of the tool.

The model further assessed the impact of social influence and facilitating conditions on aversion toward AI. The result found that both have a significant negative effect on AI aversion. This implies that when organizations create a supportive environment where seniors, peers, and subordinates use the same technology, employees are encouraged to use AI. Also, when the organizations provide supportive interventions in the form of training, resources, and other facilities, there is a low level of aversion toward AI. Past studies have shown the influence of antecedents on attitude toward technology (Cao et al., 2021; Chatterjee et al., 2021; Dwivedi et al., 2021). No model includes AI aversion in technology adoption models. Chatterjee et al. (2021) have shown the influence of facilitating conditions on attitude in customer relationship management in an Indian setting. In the context of the study, both social influence and facilitating conditions would play a role in reducing aversion. As per social identity theory, AI collaborative tools are used by team members, and if some members of the team adopt the technology, there would be a form of social pressure on others to adopt the technology, or else the team members might feel left out (Tajfel et al., 1979). While facilitating conditions have been found to be important in many previous studies, they also play an important role in the context of Indian social development organizations (Chatterjee and Bhattacharjee, 2020). The support provided by the organization to the employee in gaining knowledge about the use and benefits of these tools can help to reduce aversion toward AI.

The use of technology has a positive and significant influence on the perceived collaboration climate in an organization, thereby supporting the study’s fifth hypothesis. The use of AI-enabled technology showed a 57.2% variance in perceived collaboration in the organization. The results show that using AI-enabled tools builds collaboration among employees. These tools facilitate smooth, prompt, and easy-to-use communication channels, leading to greater employee engagement and collaboration (Frommert et al., 2018). Novel and hybrid forms of organizational designs that support work from anywhere are poised to benefit from these tools. They provide an opportunity to use technology to effectively manage team tasks and make decisions with human-AI collaboration (Haesevoets et al., 2021).

## Implication

### Theoretical Implication

First, the study contributes to the emerging literature on AI in organizations. It builds on the past conceptual studies that have highlighted the potential of AI in organizations (von Krogh, 2018; Benbya et al., 2020). This model develops a comprehensive framework to understand the adoption of AI-based tools in social development organizations. This empirical study adds to the evidence supporting AI in team tasks and management (Duan et al., 2019; Larson and DeChurch, 2020). Second, the study contributes both theoretically and practically to the literature on the role of algorithmic aversion in the use of technology (Prahl and Van Swol, 2017; Berger et al., 2020). The finding also addresses the gap in applying the concept in an organizational setting (Hou and Jung, 2021). The inclusion of AI aversion in the technology adoption model helps to bridge the gap in the literature, where affective dimensions toward technology were ignored (Zhang et al., 2021; Agogo and Hess, 2018). Some recent work on AI adoption has acknowledged and included this dimension in the research (Lin et al., 2020; Cao et al., 2021). Third, as developing countries tackle the emergence of AI, the study’s findings can be helpful in the adoption of technology. The results can be generalized to other developing countries considering the low level of awareness about AI in them. The employee’s aversion toward AI can be inhibitive in their adoption. Thus, the finding can help to create a supportive environment in social development organizations in developing countries that assist the use of AI-based tools. Fourth, the study contributes to the literature on how the use of AI influences team behavior in an organizational setup (Larson and DeChurch, 2020). Most of the past work on AI adoption in human teams has been limited to lab setups (Strohkorb Sebo et al., 2018; Zhang et al., 2021). The finding backs the role of AI in supporting team behavior. Fifth, the study shares a strong prediction of the UTAUT model in using AI-enabled tools by employees in social development organizations. The model has been extended with the inclusion of AI aversion as an exogenous factor that impacts the use. The study contributes to the literature by extending the model to evaluate the outcome of use on collaborative employee experiences, as many previous technology adoption works have limited use and intention to use technology (Venkatesh, 2021).

### Practical Implication

The proposed model would help organizations understand employees’ perspectives on how technology should be introduced with caution for antecedents and confounding factors that can help accentuate the use of technology among employees. The presented UTAUT model can precisely understand the adoption of AI-enabled tools for unified communication and collaboration among employees in the social development sector in India. This finding can be helpful at both the individual and organizational levels, as they share insights into how technology influences employees’ experiences in the organizational context. The finding of the study offers valuable managerial implications. First, organizations aspiring to adopt AI-enabled tools should be cautious of performance expectancy, effort expectancy, social influence, and facilitating conditions as they are critical antecedents influencing the use. The organization can support managers by providing enabling technical infrastructure and training by educating them about AI-based technology (Schoemaker and Tetlock, 2017; Ransbotham et al., 2018; Dwivedi et al., 2021). Building social influence with increased use of these tools by senior management can encourage the adoption of AI, as this behavior can be modeled by the subordinates, thereby encouraging organization-wide adoption.

Second, AI aversion is a critical factor that influences AI-enabled tools. An organization should be cautious about employees’ perception of AI as that affects the adoption and use of technology (Duan et al., 2019; Tambe et al., 2019; Cao et al., 2021). The model found that aversion can influence the employee’s performance expectancy and use, directly and indirectly influencing the adoption of AI-enabled tools. Organizations can help to elevate the perception of AI by building knowledge about AI (Jöhnk et al., 2021; Chowdhury et al., 2022). According to the literature, there is a decreased adoption and use of AI-enabled tools due to a lack of understanding about AI, with the perception of systems being unpredictable and dangerous (dark side) (Mahmud et al., 2022). Thus, understanding AI tools’ capabilities and their impact on team management and performance can help to reduce aversion and increase performance expectancy. This is also relevant in the Indian context and, more specifically, in the social development sector, where there is insufficient knowledge about AI and its capabilities (Chatterjee and Bhattacharjee, 2020). Third, another critical factor influencing AI-enabled tools is social influence. There can be a greater fear of being left out in teams, and people are greatly influenced by the perception of colleagues and supervisors in adopting the algorithm (Mahmud et al., 2022). Encouraging the use of AI by critical hubs and social influencers in organizations can promote the adoption of AI-based technology. There is evidence that existing and previous users can influence the willingness to adopt the algorithms of others (Alexander et al., 2018; Zhang et al., 2021). To succeed with a new form of technology such as AI, organizations must continuously learn new habits, acquire new skills, and effectively lead massive transformations (Novak et al., 2021). Finally, in developing countries such as India, the limited diffusion of technology is an inhibitor of growth and development (Iyer and Banerjee, 2015). Low technology utilization has impacted productivity and capability (Rosales et al., 2020). The study’s findings would help to guide organizations in the social development sector by referring to a specific model for adopting AI-enabled tools. Thus, for Indian capability growth, creating a supportive environment that encourages technology uptake would benefit the national buildings (Jayaraman et al., 2018).

## Limitation

Some limitations in this study should be addressed in future studies. The data obtained were only from Indian employees. Hence, generalizing these results and the usage behavior of employees in different countries and cultures can be challenging. The study was cross-sectional and conducted within a short interval of time. Over time, people’s experiences with technology can change as they accumulate knowledge and experience. A longitudinal approach to examining technology usage behavior could be more insightful. Also, self-report questionnaires were used in the study. While answering, the respondents might not share their genuine opinions, leading to response bias (Straub et al., 2002). There was no standardized scale of algorithm aversion. A unified scale would allow a better understanding of the relation of AI aversion with other variables (Mahmud et al., 2022). Future studies should explore the extended UTAUT model to specific technologies other than AI-enabled tools to help understand its application to other technology tools. There are industrial differences in technology usage that can be explored across countries.

## Conclusion

The study attempts to develop an integrated model for social development organizations that explains the antecedents of employees to use AI-enabled tools. Extending the model with the inclusion of AI aversion helps to understand its role in adopting use. The UTAUT model has been vital in identifying antecedents in adopting AI in organizations. The finding highlights that social development organizations should be cautious of their aversion toward AI. They should focus on developing a positive social influence of AI, providing better facilitating conditions to ensure a low aversion toward AI, leading to greater acceptance. The study supports the assumption that adopting AI-enabled tools will encourage better collaborative experiences for employees in organizations.

## Data Availability Statement

The raw data supporting the conclusions of this article will be made available by the authors, without undue reservation.

## Ethics Statement

Ethical review and approval was not required for the study in accordance with the local legislation and institutional requirements. The procedures followed in the study involving human participants were following the ethical standards of the institutional and/or national research committee and with the 1964 Helsinki Declaration and its later amendments or comparable ethical standards. The patients/participants provided their written informed consent to participate in this study. Written informed consent was obtained from the individual(s) for the publication of any potentially identifiable images or data included in this article.

## Author Contributions

All authors confirm their contribution to the following: study conception and design, data collection, analysis and interpretation of results, and manuscript preparation.

## Conflict of Interest

The authors declare that the research was conducted in the absence of any commercial or financial relationships that could be construed as a potential conflict of interest.

## Publisher’s Note

All claims expressed in this article are solely those of the authors and do not necessarily represent those of their affiliated organizations, or those of the publisher, the editors and the reviewers. Any product that may be evaluated in this article, or claim that may be made by its manufacturer, is not guaranteed or endorsed by the publisher.
