# Supplementary material for: Adoption of AI-Enabled Tools in Social Development Organizations in India: An Extension of UTAUT Model
Source: Front Psychol. 2022 Jun 20;13:893691. doi: 10.3389/fpsyg.2022.893691 (PMC9251489; doi:10.3389/fpsyg.2022.893691)
Supplement: Supplementary file 1 [file Table_1.docx]

**Supplementary Table 1: Measures**

| Variable | Measure | Source |
| --- | --- | --- |
| Performance Expectancy (PE) | 1. I find AI enabled tools useful for collaboration in teams 2. Using AI enabled tools enables me to have faster communication with my team 3. Using AI enabled tools increased team’s productivity 4. Using AI enabled tools increased our team performance | (Venkatesh et al., 2012)  (Martins et al., 2014) |
| Effort Expectancy (EE) | 1. Learning how to use AI enabled tools was easy for me 2. My interaction with AI enabled tools in clear and understandable 3. I find AI enabled tools easy to use 4. It is easy for me to become skillful in using AI enabled tools | (Venkatesh et al., 2012)  (Martins et al., 2014) |
| Social Influence (SI) | 1. People who are important to me think that I should use AI enabled tools 2. People who influence my behavior think that I should use AI enabled tools 3. People whose opinion that I value think I should use AI enabled tools | (Venkatesh et al., 2012)  (Martins et al., 2014) |
| Facilitating Conditions (FC) | 1. I have resources necessary to use AI enabled tools 2. I have the knowledge necessary to use AI enabled tool 3. AI enabled tools are compatible with other technologies I use 4. I can get help from others when I have difficulties using AI enabled tools | (Venkatesh et al., 2012)  (Martins et al., 2014) |
| Use Behaviour (U) | 1. I use AI enabled tools frequently for my work 2. I use many functions of the AI enabled tools 3. I depend on AI enabled tools for my work. | Ain, N., Kaur, K., & Waheed, M. (2016). |
| Collaboration (CO) | 1. While using AI enabled tools, my teammates and I provide each other with useful information that makes work progress 2. While using AI enabled tools, my teammates and I share knowledge that promotes work progress 3. While using AI enabled tools, my teammates and I understand each other when we talk about the work to be done 4. While using AI enabled tools, my teammates and I share resources that help perform tasks 5. While using AI enabled tools, my teammates and I communicate our ideas to each other about the work to be done 6. While using AI enabled tools, my teammates and I carry out our tasks at the appropriate moment 7. While using AI enabled tools, my teammates and I make sure our tasks are completed on time 8. While using AI enabled tools, my teammates and I make adjustments in order to meet deadlines 9. While using AI enabled tools, my teammates and I make progress reports 10. While using AI enabled tools, my teammates and I exchange information on ‘who does what.’ 11. While using AI enabled tools, my teammates and I discuss work deadlines with each other 12. While using AI enabled tools, my teammates and I can foresee each others’ needs without having to express them 13. While using AI enabled tools, my teammates and I instinctively reorganize our tasks when changes are required 14. While using AI enabled tools, my teammates and I have an implicit understanding of the assigned tasks | Chiocchio and Grenier (2012) |
| Algorithm Aversion | 1. Interacting with and using AI makes me uneasy 2. With Increased reliance on AI I fear that our way of life would change for the worse 3. I am afraid that our own organizational culture would be lost with increased reliance on AI 4. Our reliance on AI is out of control 5. I doubt that AI will put the interests of humans first when making decisions | Adapted scale Van Der Veer, et al. (2013) |
