# Supplementary material for: Adoption of AI-Enabled Tools in Social Development Organizations in India: An Extension of UTAUT Model
Source: Front Psychol. 2022 Jun 20;13:893691. doi: 10.3389/fpsyg.2022.893691 (PMC9251489; doi:10.3389/fpsyg.2022.893691)
Supplement: Supplementary file 2 [file Table_2.docx]

**Supplementary Table 2: Result of Discriminant Validity by the Cross Loading**

| Item | PE | EE | SI | FC | U | CO | AIav |
| --- | --- | --- | --- | --- | --- | --- | --- |
| PE1  PE2  PE3  PE4 | **0.755**  **0.727**  **0.742**  **0.756** | 0.286  0.227  0.301  0.277 | 0.321  0.279  0.291  0.311 | 0.234  0.260  0.244  0.275 | 0.531  0.522  0.537  0.603 | 0.412  0.425  0.409  0.392 | 0.541  0.498  0.503  0.523 |
| EE1  EE2  EE3  EE4 | 0.309  0.215  0.264  0.227 | **0.754**  **0.731**  **0.818**  **0.744** | 0.369  0.355  0.275  0.283 | 0.185  0.167  0.148  0.181 | 0.342  0.258  0.349  0.274 | 0.345  0.321  0.320  0.266 | 0.351  0.264  0.286  0.295 |
| SI1  SI2  SI3 | 0.245  0.267  0.322 | 0.234  0.319  0.302 | **0.815**  **0.781**  **0.795** | 0.179  0.183  0.245 | 0.287  0.338  0.353 | 0.355  0.325  0.238 | 0.309  0.288  0.321 |
| FC1  FC2  FC3  FC4 | 0.273  0.294  0.312  0.267 | 0.165  0.183  0.107  0.134 | 0.194  0.226  0.217  0.118 | **0.788**  **0.758**  **0.851**  **0.799** | 0.211  0.234  0.217  0.194 | 0.382  0.291  0.321  0.322 | 0.369  0.298  0.254  0.314 |
| U1  U2  U3 | 0.512  0.487  0.468 | 0.379  0.357  0.316 | 0.381  0.294  0.337 | 0.281  0.277  0.352 | **0.881**  **0.785**  **0.727** | 0.573  0.486  0.432 | 0.261  0.282  0.301 |
| CO1  CO2  CO3  CO4  CO5  CO6  CO7  CO8  CO9  CO10  CO11  CO12  CO13  CO14 | 0.352  0.328  0.292  0.368  0.391  0.295  0.398  0.328  0.347  0.376  0.283  0.256  0.384  0.374 | 0.162  0.268  0.188  0.149  0.179  0.239  0.156  0.244  0.238  0.103  0.136  0.194  0.155  0.259 | 0.403  0.378  0.321  0.345  0.397  0.355  0.322  0.294  0.265  0.341  0.362  0.383  0.352  0.362 | 0.147  0.193  0.183  0.221  0.108  0.197  0.136  0.148  0.189  0.203  0.154  0.139  0.122  0.207 | 0.382  0.376  0.383  0.329  0.397  0.293  0.302  0.352  0.374  0.345  0.322  0.363  0.382  0.299 | **0.817**  **0.716**  **0.789**  **0.860**  **0.748**  **0.887**  **0.751**  **0.772**  **0.718**  **0.728**  **0.872**  **0.831**  **0.741**  **0.794** | 0.532  0.428  0.423  0.472  0.429  0.463  0.521  0.459  0.448  0.437  0.484  0.498  0.504  0.473 |
| AIav1  AIav2  AIav3  AIav4  AIav5 | 0.263  0.247  0.288  0.291  0.294 | 0.139  0.176  0.163  0.199  0.132 | 0.219  0.232  0.193  0.228  0.227 | 0.563  0.483  0.429  0.432  0.477 | 0.232  0.258  0.263  0.274  0.264 | 0.300  0.352  0.321  0.372  0.392 | **0.756**  **0.825**  **0.802**  **0.741**  **0.764** |
